# ﻿A survey of pholcid spiders (Araneae, Pholcidae) from Guiyang, Guizhou Province, China

**DOI:** 10.3897/zookeys.1186.105736

**Published:** 2023-12-13

**Authors:** Lan Yang, Fangyu Zhao, Qiaoqiao He, Zhiyuan Yao

**Affiliations:** 1 College of Life Science, Shenyang Normal University, Shenyang 110034, Liaoning, China Shenyang Normal University Shenyang China; 2 Liaoning Key Laboratory of Evolution and Biodiversity, Shenyang 110034, Liaoning, China Liaoning Key Laboratory of Evolution and Biodiversity Shenyang China; 3 Liaoning Key Laboratory for Biological Evolution and Agricultural Ecology, Shenyang 110034, Liaoning, China Liaoning Key Laboratory for Biological Evolution and Agricultural Ecology Shenyang China

**Keywords:** Biodiversity, fauna, new record, new species, taxonomy

## Abstract

The family Pholcidae C.L. Koch, 1850 is highly diverse in Guizhou Province, southwestern China, and currently contains four genera and 22 species. Nevertheless, the distribution of pholcid spiders is conspicuously patchy in Guizhou. Species from Guiyang are poorly studied, and only *Pholcusspilis* Zhu & Gong, 1991 has been recorded. A survey was undertaken for the first time to study the pholcids in Guiyang. A total of four species are reported, comprising *Belisanayuhaoi* Yang & Yao, **sp. nov.** and three other species: *Leptopholcustanikawai* Irie, 1999 (new record for Guiyang), *Pholcusspilis* Zhu & Gong, 1991 and *Spermophorasenoculata* (Dugès, 1836) (new record for Guizhou).

## ﻿Introduction

The species-rich spider family Pholcidae currently contains 97 genera and 1937 species (World Spider Catalog 2023). It comprises five subfamilies: Arteminae Simon, 1893; Modisiminae Simon, 1893; Ninetinae Simon, 1890; Smeringopinae Simon, 1893; and Pholcinae C.L. Koch, 1850 (Huber 2011a; Dimitrov et al. 2013; Eberle et al. 2018), and has a worldwide distribution. Recently, a series of surveys of pholcid spiders have been undertaken in northern China. For instance, the expedition to Changbai Mountains revealed 26 species recorded from Liaoning Province (Lu et al. 2021; Yao et al. 2021; World Spider Catalog 2023; Zhao et al. 2023a). The expeditions to Yanshan-Taihang Mountains and Lüliang Mountains brought the fauna of pholcids from Hebei Province, Beijing and Shanxi Province to 31 species, six species, and 21 species, respectively (Lu et al. 2022a, b; World Spider Catalog 2023; Zhao et al. 2023b). To date, 17 genera and 271 species of pholcids have been recorded from China (World Spider Catalog 2023), of which nine genera and 99 species were collected in caves or at cave entrance ecotones from karst regions.

**Figure 1.**
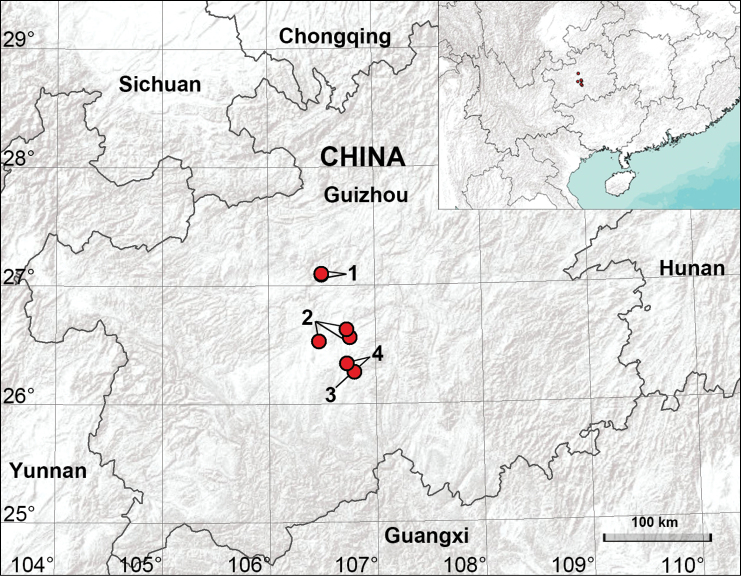
Distribution of Pholcidae treated in this paper **1***Belisanayuhaoi* sp. nov. **2***Leptopholcustanikawai***3***Pholcusspilis***4***Spermophorasenoculata*.

**Figure 2.**
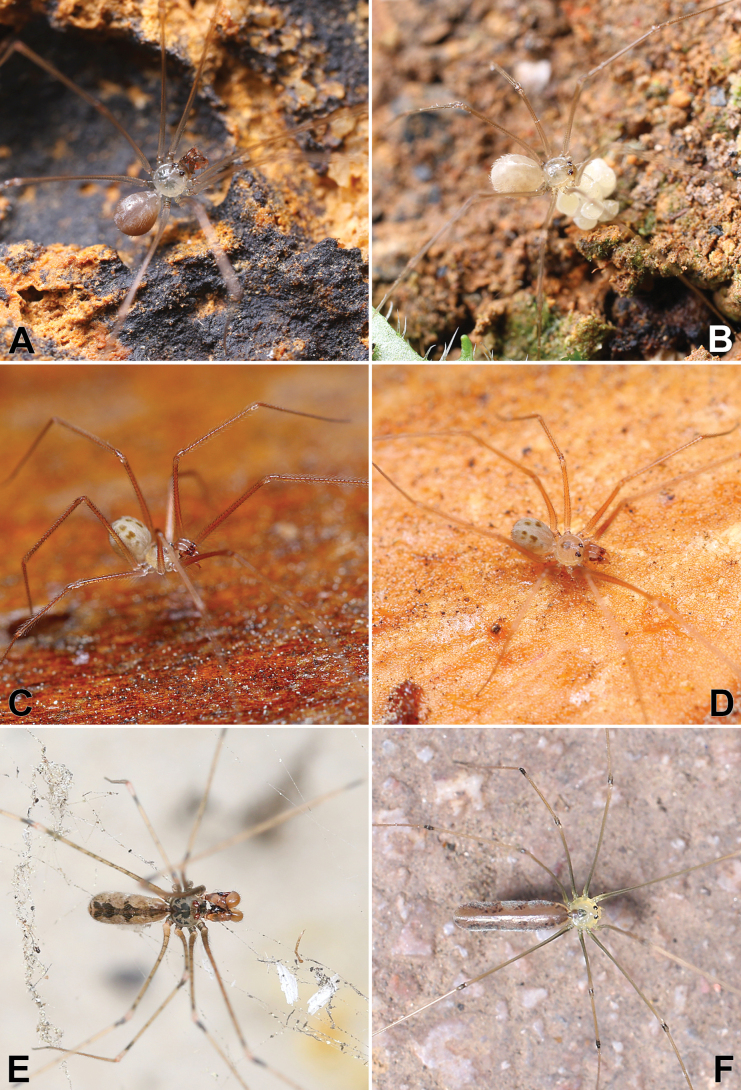
Living specimens of Pholcidae treated in this paper **A, B***Belisanayuhaoi* sp. nov. (♂♀) **C, D***Spermophorasenoculata* (♂) **E***Pholcusspilis* (♂) **F***Leptopholcustanikawai* (juvenile). Photographs by Q Lu (Shenzhen).

Guizhou Province, in the southwest of China, is one of the most spectacular examples of humid subtropical karst landscapes, and also exhibits high diversity of pholcids. Currently, four genera (*Belisana* Thorell, 1898, *Khorata* Huber, 2005, *Leptopholcus* Simon, 1893, *Pholcus* Walckenaer, 1805) and 22 species have been recorded (World Spider Catalog 2023). Nevertheless, only one species, *Pholcusspilis* Zhu & Gong, 1991, has been recorded from Guiyang, the provincial capital of Guizhou. In this paper, we undertook a survey in Guiyang for the first time and report four species, comprising a new species from a cave and three known species (Figs 1, 2).

## ﻿Material and methods

Specimens were examined and measured with a Leica M205 C stereomicroscope. The left male palp was photographed. The epigyne was photographed before dissection. The vulva was treated in a 10% warm solution of potassium hydroxide (KOH) to dissolve soft tissues before illustration. Images were captured with a Canon EOS 750D wide zoom digital camera (24.2 megapixels) mounted on the stereomicroscope mentioned above and assembled using Helicon Focus v.3.10.3 image stacking software (Khmelik et al. 2005). All measurements are given in millimeters (mm). Leg measurements are shown as: total length (femur, patella, tibia, metatarsus and tarsus). Leg segments were measured on their dorsal sides. The distribution map was generated with ArcGIS v. 10.2 (ESRI Inc. 2002). The specimens studied are preserved in 75% ethanol and deposited in the College of Life Science, Shenyang Normal University (**SYNU**) in Liaoning, China and Guizhou Normal University (**GZNU**) in Guizhou, China.

Terminology and taxonomic descriptions follow Huber (2005) and Yao et al. (2015). The following abbreviations are used in the descriptions: **ALE** = anterior lateral eye, **AME** = anterior median eye, **PME** = posterior median eye, **L/d** = length/diameter; used in the illustrations: **b** = bulb, **ba** = bulbal apophysis, **da** = distal apophysis, **e** = embolus, **ep** = epigynal pocket, **f** = flap, **pa** = proximo-lateral apophysis, **pp** = pore plate, **pr** = procursus.

## ﻿Taxonomic accounts

### ﻿Family Pholcidae C.L. Koch, 1850

**Subfamily Pholcinae C.L. Koch, 1850**

#### ﻿Genus Belisana

Taxon classificationAnimaliaAraneaePholcidae

Thorell, 1898

D1F9184B-E636-5904-BE97-7F14975DDC2D

##### Type species.

*Belisanatauricornis* Thorell, 1898.

#### ﻿ Belisana yuhaoi

Taxon classificationAnimaliaAraneaePholcidae

Yang & Yao sp. nov.

54A4991B-1E4F-5AC3-A776-572025532A9C

https://zoobank.org/35D4BC52-53E7-47D4-9E54-2FC5291E33DC

Figs 3 , 4

##### Type material.

Holotype: ♂ (SYNU-Ar00301), cave without a name (27°5.40'N, 106°30.00'E, 1109 m), Liutong Town, Xiuwen County, Guiyang, **Guizhou**, **China**, 5 June 2022, H Yu & Q Lu leg. Paratypes: 2♂ (SYNU-Ar00302, Ar00303) and 3♀ (SYNU-Ar00304–00306), same data as for the holotype. 1♀ (SYNU-Ar00307), Duobing Cave (27°6.00'N, 106°30.00'E, 1026 m), other data as for the holotype.

##### Etymology.

The specific name is a patronym in honor of the collector Hao Yu; noun (name) in genitive case.

##### Diagnosis.

**Figure 3.**
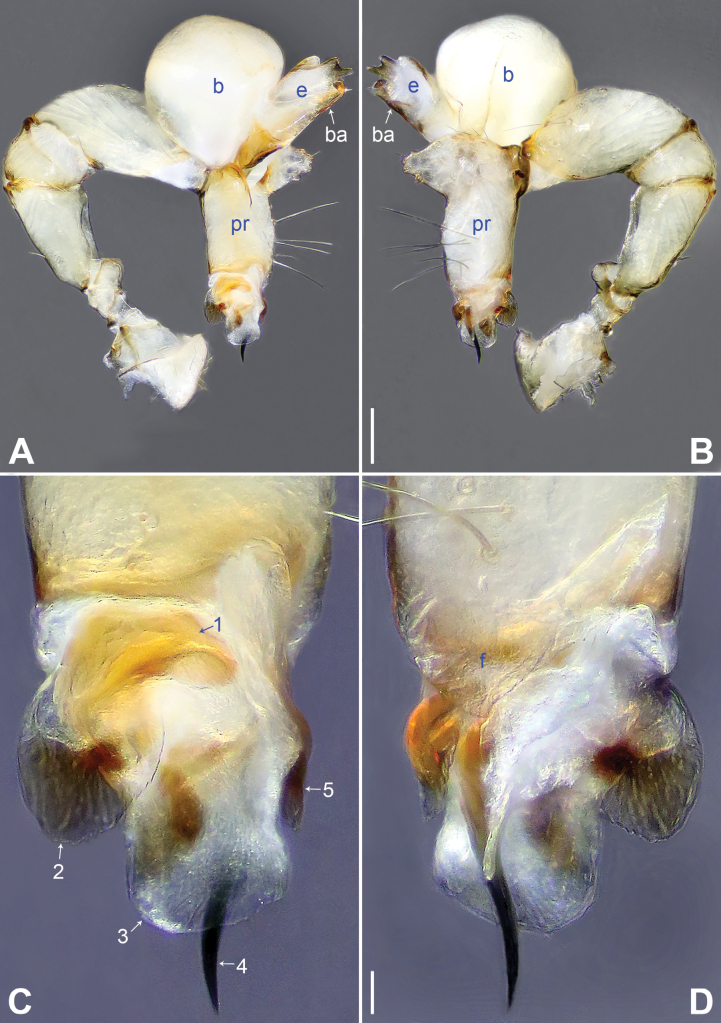
*Belisanayuhaoi* sp. nov., holotype male **A, B** palp: **A** prolateral view **B** retrolateral view **C, D** distal part of procursus: **C** prolateral view, arrow 1 indicates prolatero-subdistal sclerite, arrow 2 indicates sclerotized prolatero-ventral lamella, arrow 3 indicates distal membranous lamella, arrow 4 indicates curved distal spine, arrow 5 indicates sclerotized dorsal apophysis **D** retrolateral view. Abbreviations: b = bulb, ba = bulbal apophysis, e = embolus, f = flap, pr = procursus. Scale bars: 0.10 mm (**A, B**); 0.02 mm (**C, D**).

**Figure 4.**
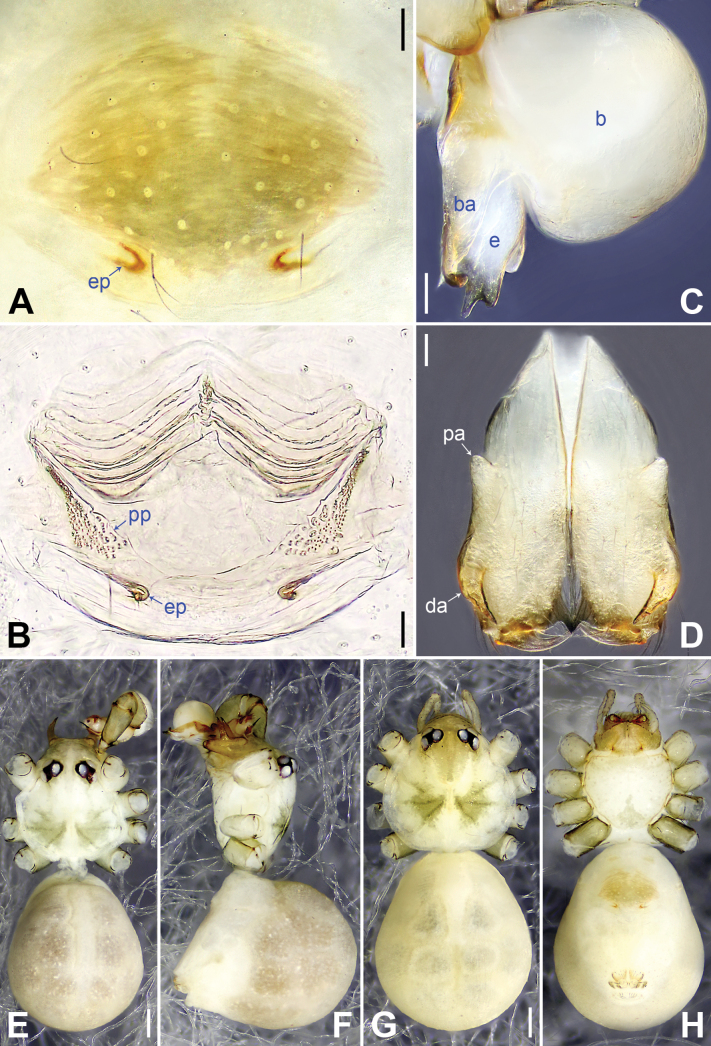
*Belisanayuhaoi* sp. nov., holotype male (**C–F**) and paratype female (**A, B, G, H**) **A** epigyne, ventral view **B** vulva, dorsal view **C** bulbal apophyses, prolateral view **D** chelicerae, frontal view **E–H** habitus: **E, G** dorsal view **F** lateral view **H** ventral view. Abbreviations: b = bulb, ba = bulbal apophysis, da = distal apophysis, e = embolus, ep = epigynal pocket, pa = proximo-lateral apophysis, pp = pore plate. Scale bars: 0.05 mm (**A–D**); 0.20 mm (**E–H**).

The new species resembles *B.galeiformis* Zhang & Peng, 2011 (Zhang and Peng 2011: 52, fig. 1A–F) with similar male chelicerae and bulbal apophysis (Fig. 4C, D), but it can be distinguished by prolatero-ventral lamella of procursus nearly round (arrow 2 in Fig. 3C; nearly angular in *B.galeiformis*), by distal membranous lamella of procursus laterally weak sclerotized (sclerotized part nearly half-round, arrow 3 in Fig. 3C; with triangular sclerite in *B.galeiformis*), by procursus with triangular retrolateral membranous flap (f in Fig. 3D; flap large and half-round in *B.galeiformis*), by epigynal plate nearly round, posteriorly strongly curved (Fig. 4A; hat-shaped, posteriorly straight in *B.galeiformis*), and by vulval pore plates nearly triangular (Fig. 4B; long elliptic in *B.galeiformis*).

##### Description.

**Male** (holotype, SYNU-Ar00301): total length 1.93 (2.03 with clypeus), prosoma 0.74 long, 0.78 wide, opisthosoma 1.19 long, 0.96 wide. Legs I and IV missing, femur II: 3.92 (other segments missing), leg III: 10.18 (2.97, 0.33, 2.48, 3.56, 0.84). Eye interdistances and diameters: PME–PME 0.13, PME 0.10, PME–ALE 0.04, AME absent. Sternum width/length: 0.65/0.62. Habitus as in Fig. 4E, F. Dorsal shield of prosoma yellowish, with large, brown radiating marks; ocular area and clypeus yellowish, with brown marks; sternum yellowish, with triangular posterior brown marks. Legs whitish, without darker rings. Opisthosoma yellowish, with dorsal and lateral brown spots. Ocular area not elevated. Thoracic furrow absent. Clypeus unmodified. Chelicerae as in Fig. 4D, with a pair of proximo-lateral apophyses and a pair of curved distal apophyses (distance between tips of distal apophyses: 0.21). Palp as in Fig. 3A, B; trochanter with short retrolatero-ventral apophysis; femur with small retrolatero-proximal protrusion; procursus simple proximally but complex distally, with prolatero-subdistal sclerite (arrow 1 in Fig. 3C), sclerotized prolatero-ventral lamella (arrow 2 in Fig. 3C), distal membranous lamella (arrow 3 in Fig. 3C), curved distal spine (arrow 4 in Fig. 3C), sclerotized dorsal apophysis (arrow 5 in Fig. 3C), and angular retrolateral membranous flap (f in Fig. 3D); bulb (Fig. 4C) with hooked apophysis and simple embolus.

**Female** (paratype, SYNU-Ar00304): similar to male, habitus as in Fig. 4G, H. Total length 2.03 (2.13 with clypeus), prosoma 0.83 long, 0.91 wide, opisthosoma 1.20 long, 1.04 wide; tibia I: 4.02; tibia I L/d: 50. Eye interdistances and diameters: PME–PME 0.12, PME 0.08, PME–ALE 0.03, AME absent. Sternum width/length: 0.58/0.51. Epigyne (Fig. 4A) simple and flat, with brown marks and a pair of posterior pockets, 0.17 apart (ep in Fig. 4A, B). Vulva (Fig. 4B) with ridge-shaped anterior arch and a pair of nearly triangular pore plates.

##### Variation.

In one male paratype (SYNU-Ar00302), leg I: 23.28 (6.02, 0.39, 5.71, 9.36, 1.80); tibia I L/d: 60. Retrolateral trichobothrium of tibia I at 6% proximally; legs with short vertical setae on metatarsi, without spines and curved setae; tarsus I with 22 distinct pseudosegments. Tibia I in another male paratype (SYNU-Ar00303): 5.38. Tibia I in the other two female paratypes (SYNU-Ar00305, Ar00306): 3.28, 3.75 (leg I missing in SYNU-Ar00307).

##### Habitat.

The species was found inside cave.

##### Distribution.

China (Xiuwen County in Guizhou; type locality, Fig. 1).

#### ﻿Genus Leptopholcus

Taxon classificationAnimaliaAraneaePholcidae

Simon, 1893

EE22726D-AD67-5EE7-A37C-74F611CD3EAA

##### Type species.

*Leptopholcussignifer* Simon, 1893.

#### ﻿ Leptopholcus tanikawai

Taxon classificationAnimaliaAraneaePholcidae

Irie, 1999

08BDBDF9-0F10-522B-84D6-CEAC43EE9E62

Leptopholcus tanikawai Irie, 1999: 37, figs 1–5 (♂♀).
Leptopholcus tanikawai Irie, 2009: 108, figs 9–11 (♂♀). Huber 2011b: 97, figs 228–231, 273, 274, 395–401, 426, 427 (♂♀). Fu and Chen 2017: 18, figs 2A–E, 3A–E (♂♀).

##### New material examined.

1♂ (GZNU), Taoyuanhe (26°31.80'N, 106°28.20'E, 1237 m), Xiuwen County, Guiyang, **Guizhou**, **China**, 4 June 2022, H Yu leg. 3♀ (GZNU), a forest park (26°33.60'N, 106°45.60'E, 1165 m), Nanming District, Guiyang, **Guizhou**, **China**, 10 August 2021, H Yu, H Zhang, D Wang, L Li & J Xin leg. 1 juvenile (GZNU), Guizhou Botanical Garden (26°37.80'N, 106°43.80'E, 1249 m), Yunyan District, Guiyang, **Guizhou**, **China**, 4 June 2022, H Yu & Q Lu leg.

##### Distribution.

China (Xiuwen County, Nanming District and Yunyan District in Guizhou; Fig. 1).

#### ﻿Genus Pholcus

Taxon classificationAnimaliaAraneaePholcidae

Walckenaer, 1805

94A8641A-9372-5AC5-94D4-C15EF8F1C0A7

##### Type species.

*Araneaphalangoides* Fuesslin, 1775.

#### ﻿ Pholcus spilis

Taxon classificationAnimaliaAraneaePholcidae

Zhu & Gong, 1991

882C420C-69F6-501F-9249-25DCD80A61ED

Pholcus spilis Zhu & Gong, 1991: 22, fig. 4A–G (♂♀).
Pholcus spilis Song, Zhu and Chen 1999: 59, fig. 24E–H (♂♀). Zhang and Zhu 2009: 83, fig. 47A–G (♂♀). Huber 2011b: 359, figs 1654, 1727, 1728 (♂). Yao and Li 2012: 33, figs 161A–D, 162A–C (♂♀). Yin et al. 2012: 171, fig. 35a–g (♂♀). Zhang 2018: 4, figs 1–3A–F, pl. 1 (♂♀).

##### New material examined.

1♂ (GZNU), Sanchahe (26°16.20'N, 106°48.00'E, 1162 m), Gaopo Town, Huaxi District, Guiyang, **Guizhou**, **China**, 20 May 2022, H Yu & Q Lu leg.

##### Distribution.

China (Huaxi District in Guizhou; Fig. 1).

#### ﻿Genus Spermophora

Taxon classificationAnimaliaAraneaePholcidae

Hentz, 1841

DA53F072-2ACB-59FE-BFB4-7550C5C8E340

##### Type species.

*Spermophorameridionalis* Hentz, 1841.

#### ﻿ Spermophora senoculata

Taxon classificationAnimaliaAraneaePholcidae

(Dugès, 1836)

0E64A29C-1C55-5659-9C1B-D54585750AA1

See World Spider Catalog 2023

##### New material examined.

2♂ (GZNU) and 2♀ (GZNU), Sanchahe (26°16.20'N, 106°48.00'E, 1162 m), Gaopo Town, Huaxi District, Guiyang, **Guizhou**, **China**, 20 May 2022, H Yu & Q Lu leg. 1♂ (GZNU), Laobanghe (26°20.40'N, 106°43.80'E, 1022 m), Qiantao Town, Huaxi District, Guiyang, **Guizhou**, **China**, 18 May 2022, H Yu & Q Lu leg.

##### Distribution.

China (Huaxi District in Guizhou; Fig. 1).

## Supplementary Material

XML Treatment for Belisana

XML Treatment for Belisana yuhaoi

XML Treatment for Leptopholcus

XML Treatment for Leptopholcus tanikawai

XML Treatment for Pholcus

XML Treatment for Pholcus spilis

XML Treatment for Spermophora

XML Treatment for Spermophora senoculata

## ﻿Additional information

### Author ORCIDs

Lan Yang https://orcid.org/0000-0002-7754-9275

Fangyu Zhao https://orcid.org/0000-0002-1005-8471

Qiaoqiao He https://orcid.org/0000-0002-9381-7444

Zhiyuan Yao https://orcid.org/0000-0002-1631-0949

### Data availability

All of the data that support the findings of this study are available in the main text.
